# Multiple cysticerci as an unusual cause of mesenteric lymph node enlargement: a case report

**DOI:** 10.1186/1752-1947-2-196

**Published:** 2008-06-06

**Authors:** Harsh Mohan, Amanjit Bal, Rakhi Aulakh

**Affiliations:** 1 Government Medical College, Sector-32A, Chandigarh-160 030, India

## Abstract

**Introduction:**

Cysticercosis is a disease caused by infestation with the larval stage of the intestinal cestode *Taenia solium*. The parasite usually localizes to subcutaneous tissues and muscles causing palpable or visible nodules, to the brain leading to epileptic attacks, and to the eyes with visible nodules leading to blindness and atrophy.

**Case presentation:**

Here we present the case of a 15-year-old girl who was incidentally detected as having mesenteric lymph node enlargement caused by multiple cysticerci. This is the second case report of lymph node enlargement due to cysticercus infestation.

**Conclusion:**

This rare mode of presentation of cysticercus infestation highlights the importance of parasites as a cause of treatable lymph node enlargement.

## Introduction

Cysticercosis is a disease caused by infestation with the larval stage of the intestinal cestode *Taenia solium *that occurs when humans become intermediate hosts. It is endemic in countries where raw or undercooked pork is consumed. Although no tropism for any tissue is known to date, the parasite has a marked tendency to localize in the subcutaneous tissues and muscles causing palpable or visible nodules, in the brain leading to epileptic attacks and in the eye where it often leads to blindness and atrophy of the eye [1-3].

Here we report the case of a 15-year-old girl whose mesenteric lymph node enlargement because of multiple cysticerci was detected incidentally. This is the second case report of lymph node enlargement due to cysticercus infestation.

## Case presentation

A 15-year-old girl presented to the surgery unit with pain in the right hypochondrium and underwent appendicectomy for acute appendicitis with paralytic ileus. At laparotomy an enlarged mesentric lymph node was noted which was excised and sent for histopathological examination together with the appendicectomy specimen.

**Figure 1:**
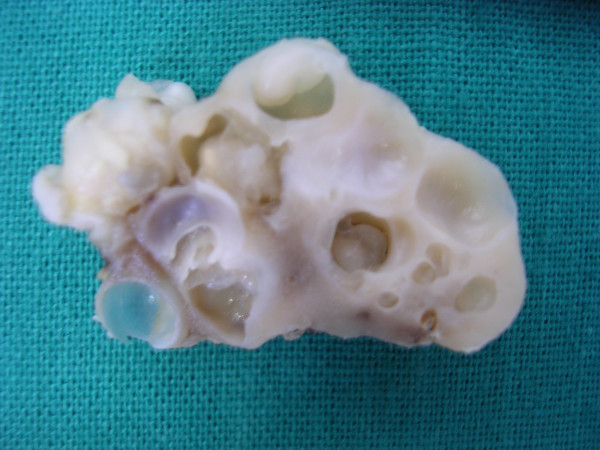
Gross photograph of the cut section of lymph node specimen showing multiple cysts, some containing shiny grey-white parasites.

**Figure 2:**
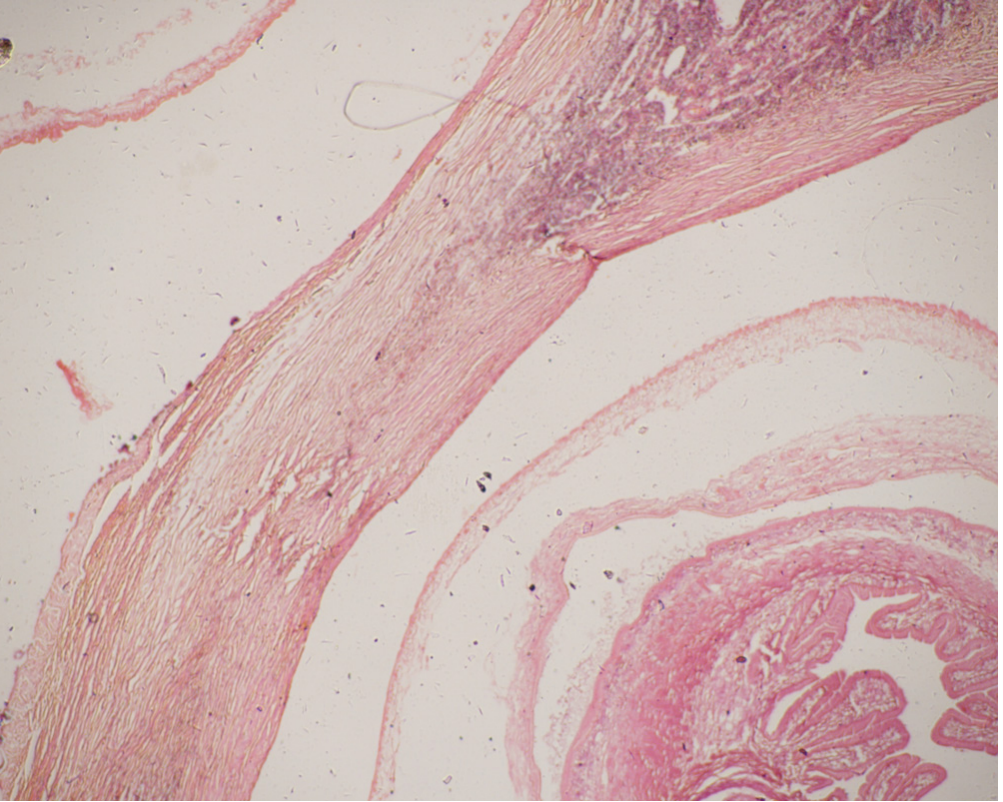
**Photomicrograph showing parts of two cysticerci with intervening lymphoid tissue**. Hematoxylin and eosin stain, 2× magnification.

Grossly, the excised mesenteric lymph node measured 2.5×1.5×1 cm. It was nodular on external examination. A cut section revealed multiple cysts varying in size from 0.5 to 0.8 cm in diameter (Figure 1) containing shiny grey-white material. Microscopic examination showed numerous parasites with characteristic morphology scattered throughout the node (Figure 2). The parasites had an outer vesicular wall composed of three layers: an outer cuticular layer, a middle cellular layer and an inner reticular layer along with invaginated scolices which showed a rudimentary body with spiral canal. Some of these parasites were surrounded by host reaction in the form of palisaded histiocytes, fibrosis and calcification in places. The appendicectomy specimen measured 4×1 cm and showed changes of acute diffuse suppurative appendicitis.

Subsequent to the histopathology report the patient underwent a further thorough physical examination that revealed no evidence of subcutaneous or muscular swelling. Further radiological and other investigations did not reveal cysticercus infestation anywhere else in the body. We were unable to confirm that the parasite was *T. solium *by serology, staining of the tissue by immunofluorescence assay or immunohistochemistry.

## Discussion

The present case is unique in that the patient presented with symptoms which were unrelated to infestation by the parasite. Furthermore, no other organs were found to be involved. It is postulated that the larvae, after evagination in the small intestine, penetrated the mesenteric lymphatics, burrowing through the bowel wall, hence reaching the mesenteric lymph node. Serology or other investigations such as Western blot, which were not available in this case, would help to identify the parasite.

Only one other case of lymph node enlargement due to cysticercus infestation in a human has been found in a search of the literature. This was the case of a 7-year-old girl who presented with seizures and submandibular lymph node enlargement. Fine needle aspiration cytology of the lymph node revealed cysticercus infestation. Other investigations showed inflammatory granulomas in the brain and the presence of anticysticercal antibodies in the serum and cerebrospinal fluid [4].

An unusual presentation of cysticercus infestation which has been reported in literature is giant cysticercosis or tumoral cysticercosis, where the larvae attain large sizes [5]. An anecdotal case of congenital neurocysticercosis due to transplacental transmission of cysticerci has also been reported [6]. A bizarre case of infestation after drinking a mixture prepared from segments passed in feces is also on record. This patient had extensive involvement of the musculature system and brain and eventually succumbed to the illness [7].

*T. solium *has a complex life cycle requiring two hosts. Humans (the definitive hosts) are infected by eating raw or undercooked 'measly' pork containing the larvae, called cysticerci. In the small intestine of man, the larvae attach to the gut wall with the help of suckers and in a few weeks grow into adult worms. They may remain here without causing any symptoms, or may be responsible for vague abdominal discomfort or intestinal disorders and anemia. The gravid terminal proglottids of the adult worm detach and are eliminated in the feces.

Pigs (the usual intermediate host) are infected by ingesting contaminated food and water. Eggs rupture in the alimentary canal of the pig releasing onchospheres, which penetrate the gut wall to gain entrance into the portal vessels or mesenteric lymphatics, finally reaching the systemic circulation. The onchospheres are filtered out of the circulating blood into the musculature system where, after undergoing further development, they remain dormant until ingested by a human [1].

A far more dangerous sequence occurs when humans act as the intermediate host. Humans can be infected by ingesting fecally contaminated food and water or through auto-infestation. The eggs hatch in the small intestine and cross the bowel wall and lodge in human tissues in the same way as occurs in pigs. Viable cysticerci do not elicit inflammatory changes in the surrounding tissues. However, when they die they can release substances that provoke an inflammatory response. Eventually the cysticerci calcify and can be seen on X-ray. The organs most commonly affected by cysticercosis are the eye (13% to 46%), subcutaneous tissue (24.5%) and brain (13.6%) [1-3].

## Conclusion

This rare mode of presentation of cysticercosis highlights the importance of parasites as a cause of treatable lymph node enlargement.

## Competing interests

The authors declare that they have no competing interests.

## Consent

Written informed consent was obtained from the patient's next-of-kin for publication of this case report and accompanying images. A copy of the written consent is available for review by the Editor-in-Chief of this journal.

## Authors' contributions

HM participated in the histopathological diagnosis, photography (gross specimen photograph as well as photomicrography) and editing the manuscript. AB participated in the histopathological diagnosis and editing of the manuscript. RA participated in the histopathological diagnosis, writing of the manuscript and photomicrography. All authors read and approved the final manuscript.
